# The excitatory neurotransmitter glutamate and its receptors: the culprits behind neuropathic pain?

**DOI:** 10.3389/fnagi.2026.1951251

**Published:** 2026-09-11

**Authors:** Pengfei Qi, Zhongren Sun, Hongna Yin

**Affiliations:** 1 Heilongjiang University of Chinese Medicine, Harbin, China; 2 The Second Affiliated Hospital of Heilongjiang University of Chinese Medicine, Harbin, China

**Keywords:** central sensitisation, glutamate, neuropathic pain, neurotransmitter, nociceptor, pain, peripheral sensitisation

## Abstract

Human understanding of the nature of pain has undergone a profound shift from mechanical reductionism to a multidimensional, integrative approach. Among the many types of chronic pain, neuropathic pain (NP) has long been a key focus of research in the fields of neurobiology and pain medicine, owing to its high prevalence and complex mechanisms. Its core pathological feature is an abnormal increase in neuron excitability caused by peripheral and central sensitization. As the most important excitatory neurotransmitter in the nervous system, glutamate plays an irreplaceable and central role in signal transduction at every level of the pain transmission pathway. Therefore, investigating the mechanisms of analgesic effects mediated by glutamate and its receptors is of great significance for understanding the pathophysiological nature of NP. This paper focuses on the mechanisms of peripheral and central sensitisation mediated by glutamate and its receptors, exploring their potential effects as key analgesic targets in NP, with a view to providing a theoretical foundation and directions for clinical translation in research into the analgesic mechanisms of NP.

## 1 Introduction

Human understanding of pain is often one-sided, with tissue damage commonly regarded as a precursor to pain. The International Association for the Study of Pain (IASP), whose mission is the study of pain, defines pain as: an unpleasant sensory and emotional experience associated with, or resembling, actual or potential tissue damage (Raja et al., 2020). The introduction of this concept not only acknowledges the multidimensional nature of pain, but also highlights its complexity more clearly. Pain may still be present even when tissue damage is imperceptible. However, the fundamental question of whether pain is essentially a psychological concept or a physiological indicator remains unresolved. As research has progressed, the revised International Classification of Diseases (ICD-11), adopted by the World Health Organisation in 2019, included a classification for chronic pain for the first time, signifying that chronic pain was recognised as a disease for the first time. At the same time, this formally signalled that pain medicine is shifting from the traditional linear ‘tissue damage–pain’ model towards a multidimensional, independent pathological condition (Arendt Nielsen et al., 2023).

As it lacks the acute warning function associated with physiological pain, chronic pain is defined as a pattern of pain that persists beyond the normal healing period (typically, pain that persists or recurs for more than 3 to 6 months) (Kang et al., 2023). Chronic pain places a huge burden on both individuals and society. According to statistics (The Lancet, 2021), over 30 per cent of the world’s population suffers from chronic pain. Depending on the aetiology, pathophysiological mechanisms and site of origin, chronic pain is classified into the following seven main categories: (1) chronic primary pain; (2) chronic cancer pain; (3) chronic post-traumatic and post-operative pain; (4) chronic neuropathic pain (NP); (5) chronic headache and orofacial pain; (6) chronic visceral pain; (7) chronic musculoskeletal pain (Treede et al., 2015). Given the wide variety of chronic pain conditions, which makes it difficult to cover them all comprehensively, this paper focuses primarily on NP, a representative type of chronic pain for which the underlying mechanisms have been relatively well studied. NP is a disease that represents a global burden. Its symptoms include spontaneous and stimulus-induced pain. It affects the peripheral nervous system and the entire central nervous system—including the spinal cord and brain regions—by mediating peripheral nociceptive sensitisation and central sensitisation (Campbell and Meyer, 2006; Bannister et al., 2020).

The onset of pain begins with the activation of nociceptors. Glutamate, as the primary neurotransmitter in sensory afferents of the nervous system, is widely distributed in both peripheral and central afferent fibres and plays a key role in the transmission of nociceptive signals (Donnelly et al., 2020). Almost all primary sensory neurones release glutamate and express its transporters and receptors. Furthermore, the vast majority of excitatory neurons in the cerebral cortex are glutamatergic neurons (Temmermand et al., 2022). The mechanisms underlying the pathophysiology of NP are, broadly speaking, still attributed to peripheral and central sensitisation, leading to an abnormal increase in neuronal excitability (Sandkühler, 2009). Abnormally elevated levels of glutamate and its binding to transporters and receptors are key factors contributing to the exacerbation of NP. Consequently, exploring how to inhibit the synthesis and release of glutamate and reduce its binding to transporters and receptors—thereby suppressing peripheral and central sensitization—represents a key mechanism for analgesic effects in NP.

This article summarises the findings of basic research conducted over the past 5 years into NP mediated by glutamate and its receptors. It aims to elucidate the mechanisms underlying the potential analgesic effects of glutamate signalling in the context of NP. The aim is to provide readers with a quick introduction to the field of pain research, whilst also offering a reliable theoretical foundation for future clinical applications.

## 2 Methods and literature search strategies

A comprehensive literature search was conducted using the PubMed, Web of Science and Google Scholar databases. The search strategy combined keyword combinations such as ‘Pain’, ‘Neuropathic pain’, ‘Glutamic acid’, ‘Nociceptors’, ‘Dorsal Root Ganglion’, ‘Dorsal Horn’, ‘Brain Region’, ‘Central Sensitisation’, ‘Peripheral Sensitisation’, ‘Animals’, and ‘Experiment’. The search covered publications from the inception of these databases up to 2026. We paid particular attention to recent research findings, especially those from the past 5 years, to ensure that the cited studies were contemporary and forward-looking. During the literature screening process, we first conducted a preliminary screening of the search results by reviewing titles and abstracts to exclude articles that were clearly irrelevant or of poor quality. For studies meeting the preliminary screening criteria, we conducted a detailed review of the full text to ensure that the included studies provided valuable insights into the topic of this review.

## 3 The physiological basis of the glutamate system: from neurotransmitter to pain mediator

**Figure 1:**
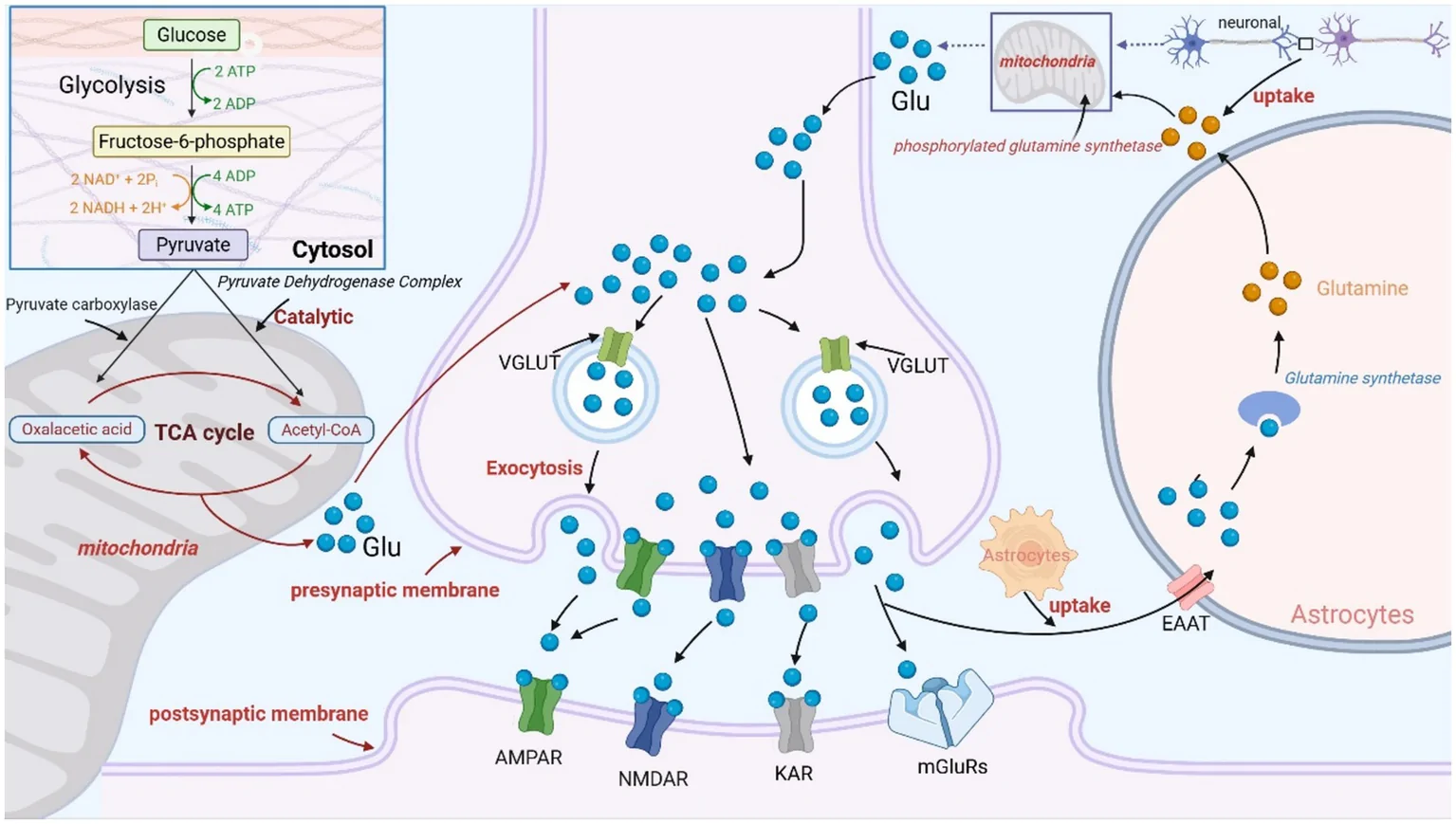
The synthesis, release and clearance of glutamate.

Glutamate is the most abundant excitatory neurotransmitter in the mammalian central nervous system; maintaining glutamate homeostasis not only ensures the normal transmission of neural signals but also prevents excitotoxicity (Watkins and Jane, 2006; Nakaya et al., 2023). Within the nociceptive pathway, the encoding and transmission of nociceptive information between neurons at all levels—from the dorsal root ganglion (DRG) to the dorsal horn of the spinal cord, the thalamus and the cortex—depend on glutamatergic signalling (Xu et al., 2008; Muqeem et al., 2018; Pinto et al., 2008; Bathel et al., 2018; Liu et al., 2025; Tiwari et al., 2013). Therefore, understanding the physiological basis of the glutamate system is a prerequisite for elucidating the mechanisms of central and peripheral sensitisation in NP. The synthesis, release and clearance of glutamate are shown in Figure 1.

### 3.1 Synthesis, release and clearance of glutamate

The synthesis of glutamate relies primarily on two precursors: glucose and glutamine; of these, the glutamine pathway is particularly crucial for maintaining the glutamate pool required for neurotransmitter function. Glutamate released into the synaptic cleft is taken up by glial cells, where it is converted into glutamine by glial-specific glutamine synthetase. The resulting glutamine is then released from the cells, taken up by neurons, and converted back into glutamate within the neuronal mitochondria by the action of phosphorylated glutamine synthetase (Bak et al., 2006; Bowtell and Bruce, 2002; Waagepetersen et al., 2005; Petroff, 2002). On the other hand, glucose is converted into pyruvate via glycolysis. Pyruvate enters the mitochondria, where it is converted into acetyl-CoA under the catalysis of the pyruvate dehydrogenase complex. At the same time, pyruvate can also be converted into Oxaloacetic acid by the action of pyruvate carboxylase. These metabolic products ultimately give rise to glutamate via the tricarboxylic acid cycle (Fillenz, 1995; Olsen and Sonnewald, 2015).

The process of glutamate release involves depolarisation, an increase in intracellular calcium ion concentration and vesicular exocytosis; that is, it exhibits the characteristics of classical neurotransmitter release (Schousboe and Waagepetersen, 2006). Within the presynaptic neuron, glutamate stored in synaptic vesicles is packaged into vesicles by vesicular glutamate transporters (VGLUT1–3). When the presynaptic neuron is stimulated, packaged glutamate is released into the synaptic cleft via exocytosis in a calcium-dependent manner during depolarisation. The released glutamate further activates postsynaptic ionotropic and metabolic receptors; this excessive stimulation mediates excitatory neurotransmission by causing harmful neuronal calcium overload (Dorsett et al., 2017; Greenamyre and Porter, 1994). Glutamate uptake has been shown to be the only mechanism capable of clearing extracellular glutamate. Through the action of VGLUTs and excitatory amino acid transporters (EAAT1–5), glutamate released from the presynaptic membrane is taken up by astrocytes or released into the extrasynaptic space, thereby preventing its binding to glutamate receptors on the postsynaptic membrane. This reduces the concentration of glutamate in the synaptic cleft, thereby terminating the transmission of excitatory signals (Marx et al., 2015; Danbolt et al., 2016). Clinical studies have found that downregulation of EAAT2 leads to elevated plasma glutamate levels, which is a key mechanism underlying chronic NP following herpes zoster. Drugs administered orally or by injection that enhance EAAT2 expression or function may potentially reduce plasma glutamate levels, block binding to postsynaptic glutamate receptors, and prevent excitotoxic damage, thereby alleviating NP (Lu et al., 2025). This suggests that upregulating EAAT2 expression in astrocytes can effectively inhibit their excessive activation, enhance glutamate clearance capacity, and consequently alleviate pain sensitisation.

### 3.2 Distribution and function of ionotropic and metabotropic glutamate receptors

Signalling in excitatory neurons in the brain is primarily mediated by glutamate. Glutamate released at synapses can activate a variety of glutamate receptors, which broadly fall into two categories: ionotropic and metabotropic (or G protein-coupled) receptors (Mayer and Armstrong, 2004; Wollmuth and Sobolevsky, 2004). Ionotropic glutamate receptors include the N-methyl-D-aspartate receptors (NMDARs), α-amino-3-hydroxy-5-methyl-4-isoxazolepropionic acid receptors (AMPARs) and Kainate receptors (KARs). These are widely distributed across the presynaptic and postsynaptic cell membranes of the central nervous system; they are responsible for the fastest excitatory signalling in the brain and are thought to contribute to synaptic plasticity (Madden, 2002). Consistent with the physiological importance of ionotropic glutamate receptors, their dysfunction is associated with a range of neuropathologies, including epilepsy (Mihály, 2019), stroke (Niu et al., 2018), and pain perception (Jergova et al., 2025). Glutamate released at synapses acts primarily on AMPARs and KARs, thereby triggering rapid excitatory synaptic transmission throughout the somatosensory nociceptive pathway. Furthermore, glutamate also binds to NMDARs, which possess voltage-dependent Mg^2+^-blocking properties, mediating slow excitatory synaptic transmission; however, this process is contingent upon AMPARs and KARs being continuously activated by glutamate, leading to intense depolarisation of the postsynaptic neuron, thereby releasing NMDARs from Mg^2+^ blockade and activating them (Xie et al., 2023; Pușcașu et al., 2024).

Metabotropic glutamate receptors (mGluRs) are G protein-coupled receptors activated by glutamate binding. They are widely distributed throughout the peripheral and central nervous systems, play a key role in neuronal excitability and synaptic transmission, and mediate slower neuromodulatory responses to glutamate (Magi et al., 2019). Studies have shown (Mazzitelli et al., 2022) that mGluRs can also mediate pain states through interactions with neuroimmune signalling. To date, eight distinct mGluR subunits have been identified and classified into Groups I–III based on their sequence homology, signal transduction pathways and drug selectivity. Group I consists of the mGluR1 and mGluR5 subunits; Group II comprises mGluR2 and mGluR3; and Group III consists of the mGluR4, mGluR6, mGluR7 and mGluR8 subunits (Tapiero et al., 2002). Furthermore, mGluR subtypes form heterodimers through specific and preferential assembly during expression; this is the primary cause of the molecular diversity and complexity of mGluRs (Lee et al., 2020; McCullock and Kammermeier, 2021). mGluRs activate intracellular signalling pathways by coupling with various G proteins (such as Gq/11 and Gi/o), thereby enabling complex and precise regulation of the nervous system (Antal, 2025). Studies have shown that the coupling of mGluRs with G proteins is a key mechanism in the regulation of pain signalling (Pomierny-Chamioło et al., 2014). mGluR1 and mGluR5 primarily exert excitatory effects through coupling with Gq, whilst the remaining mGluR subunits exert inhibitory effects through coupling with Gi/o (Su et al., 2022; Bai et al., 2023). Upon coupling with Gq, mGluR1 and mGluR5 activate phospholipase C and its downstream signalling cascades, enhancing neuronal excitability and inducing central sensitisation, thereby promoting the transmission of pain signals (Tian et al., 2024). mGluR2, mGluR3, mGluR4, mGluR6, mGluR7 and mGluR8, on the other hand, primarily couple to Gi/o. By inhibiting adenylate cyclase, reducing cyclic adenosine monophosphate (cAMP) levels, and suppressing the release of presynaptic glutamate as well as the function of glutamate receptors on primary sensory neurons, they thereby inhibit the transmission of pain signals (Xie et al., 2023; Domin and Burnat, 2024; Li et al., 2022). The coupling of mGluRs to Gq and Gi/o determines the body’s sensitivity to noxious stimuli and makes mGluRs highly promising targets for pain treatment.

### 3.3 Glutamatergic pathways in the transmission of physiological pain

Physiological pain transmission follows a typical three-stage neuronal pathway. Specifically, primary afferent fibres transmit noxious signals from the periphery via the dorsal root to the dorsal horn of the spinal cord; secondary projection neurons relay these noxious signals ascending to the thalamus; and tertiary neurons further project the noxious signals to the cerebral cortex, where the sensation of pain is perceived (Inquimbert et al., 2012). Glutamate, as the central element in each of the aforementioned stages of signal transduction, establishes a complete framework for pain transmission—from the molecular level to the circuit level—through precise metabolic regulation and a diverse distribution of receptors.

In the peripheral nervous system, noxious stimuli are converted into action potentials by the activation of nociceptors on the terminals of Aδ or C fibres, which are then conducted along the fibres. When action potentials are conducted to the presynaptic terminals in the dorsal horn of the spinal cord, they trigger the release of excitatory neurotransmitters—such as glutamate, substance P, calcitonin gene-related peptide and ATP—from the presynaptic membrane (Basbaum et al., 2009; Todd, 2010). These neurotransmitters bind to pain receptors on the postsynaptic membranes of second-order neurons in the dorsal horn of the spinal cord, thereby inducing central sensitisation (Bardoni, 2013). Secondary neurons activated in the dorsal horn of the spinal cord exhibit a quantitative or progressive response to stimulus intensity, known as the wide dynamic range (WDR) response, respond to both non-noxious light touch and noxious pain stimuli, and are therefore regarded as the common neural basis for allodynia and central sensitisation (Berta et al., 2023; Berta et al., 2017). As the central integrative hub for the processing of pain information, under physiological conditions, WDR neurons precisely encode stimulus intensity through their firing rate and integrate multiple sensory inputs, providing quantitative signals for the brain’s perception of pain; simultaneously, they integrate peripheral afferents with descending regulatory commands from the brainstem to regulate whether pain information is transmitted upwards (Fujiwara et al., 2024). However, in a neuropathological state, WDR neurons, due to abnormally increased excitability, transform into amplifiers of central sensitisation, driving the onset of allodynia and hyperalgesia (Zhang et al., 2024). Subsequently, the activated WDR neurons transmit sensory information along the contralateral anterolateral quadrant of the spinal cord, via the spinothalamic tract and other ascending pathways, to the thalamus and cortex, giving rise to the conscious perception of the pain signal. The brainstem transmits pain signals from the thalamus and the cortex to the dorsal horn of the spinal cord; by modulating the function and activity of the brain’s descending inhibitory system, it increases the body’s sensitivity to pain (Yang L. et al., 2023; Yang et al., 2024; Falk and Dickenson, 2014). Thus, the conduction of pain signals in both the peripheral and central nervous systems is realised. An understanding of the glutamatergic pathways involved in physiological pain transmission is a prerequisite for further investigation into how the glutamate system participates in and mediates peripheral and central sensitisation in pathological conditions.

## 4 Multidimensional diffusion of glutamate in NP

**Figure 2:**
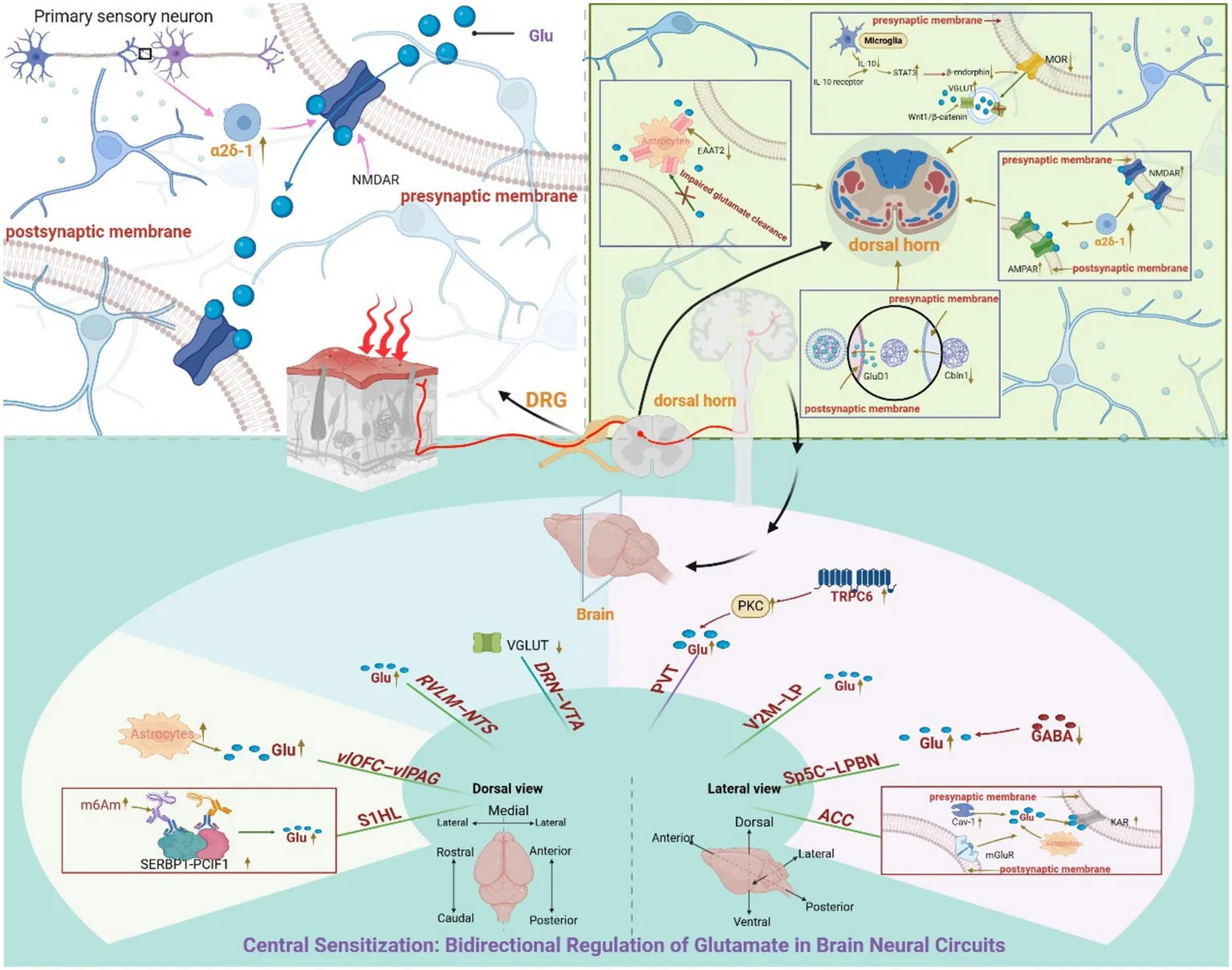
The multidimensional spread of glutamate within the pain pathways of NP.

The onset of NP is not a simple random process determined solely by electrical signals or action potentials; rather, it depends on a series of directed, multi-step events involving glutamatergic signalling along the nociceptive pathway from the DRG to the dorsal horn of the spinal cord and on to brain regions. This ultimately forms a complex regulatory network encompassing the DRG, the dorsal horn of the spinal cord and multiple brain regions. The Multidimensional Diffusion of Glutamate in NP is illustrated in Figure 2.

### 4.1 Peripheral sensitisation: abnormal excitation of glutamatergic neurons in the dorsal root ganglion

The core mechanism of peripheral sensitisation in NP lies in the abnormal excitation of glutamatergic neurons in the DRG, which accelerates the release of glutamate (Gong et al., 2014). This is not only the central mechanism of peripheral sensitisation in NP, but also a prerequisite for the occurrence of central sensitisation. Specifically, the use of optogenetics to specifically inhibit the discharge of glutamatergic neurons within the DRG reduces noxious synaptic transmission and affects the synaptic connections between neurons in the spinothalamic tract; by inhibiting the transmission of pain signals to the brain, this produces an analgesic effect on NP (Islam et al., 2025a). As a co-subunit of the voltage-gated calcium channel (VGCC), calcium voltage-gated channel auxiliary subunit alpha2delta 1 (α2δ-1) is also a direct target of gabapentin-like drugs. Studies have shown (Huang et al., 2024; Deng et al., 2019a) that upregulation of α2δ-1 expression in primary sensory neurons promotes glutamate release mediated by presynaptic NMDARs through binding to NMDARs, thereby inducing pain sensitisation. Furthermore, in DRG neurons of rats in a postherpetic neuralgia (PHN) model established by a single intraperitoneal injection of rituximab, upregulated α2δ-1 protein expression, by enhancing binding to the GluN1 in the dorsal horn of the spinal cord, leads to sustained activation of presynaptic NMDARs in the dorsal horn, thereby amplifying glutamatergic signals from Aδ fibres and ultimately inducing pain sensitization; furthermore, inhibiting glutamatergic afferent fibres in the DRG effectively alleviates pain sensitization (Huang et al., 2022).

### 4.2 Central sensitisation: glutamate release in the dorsal horn of the spinal cord, receptor aggregation and clearance

The release of glutamate in the dorsal horn of the spinal cord, along with receptor aggregation and clearance, is one of the key mechanisms mediating central sensitisation in NP. Various signalling pathways mediate central sensitisation and contribute to the development of NP by regulating glutamate release, receptor aggregation and clearance in the dorsal horn of the spinal cord. Among these, downregulation of EAAT2 in astrocytes in the dorsal horn of the spinal cord leads to impaired glutamate clearance, which in turn induces sustained central sensitization by enhancing excitatory signal transmission (Liu et al., 2024). The Wnt1/β-catenin signalling pathway enhances glutamate release by upregulating the expression of VGLUT2 in the dorsal horn of the spinal cord, thereby promoting central sensitisation and pain behaviour (Zhang et al., 2020). Other studies have shown that increased expression of VGLUT1 in the dorsal horn of the spinal cord leads to pain sensitisation by inducing abnormal budding of primary afferent fibres and dysfunction of inhibitory circuit (Li et al., 2026). Conversely, the IL-10/β-endorphin signaling pathway serves as a key bridge between the immune system and synaptic plasticity. Upon activation, microglia synthesize and secrete large amounts of IL-10; the subsequently released IL-10 binds to the microglia’s own IL-10 receptors via an autocrine mechanism and, by activating the STAT3 signaling pathway, promotes the synthesis and release of β-endorphin (Belo et al., 2023; Wu et al., 2017). β-endorphin primarily activates the μ-opioid receptor (MOR) on the presynaptic membrane of primary afferent neurons in the dorsal horn of the spinal cord. By inhibiting voltage-gated calcium channels and reducing Ca^2+^ influx, it blocks the vesicular release of glutamate. This inhibits the transmission of pain signals from primary afferent neurons to secondary neurons in the dorsal horn of the spinal cord, thereby effectively alleviating central sensitization (Ma et al., 2021).

Under physiological conditions, α2δ-1 is expressed at both presynaptic and postsynaptic sites in the dorsal horn of the spinal cord (Bonifacino et al., 2022). As a key interacting protein, α2δ-1 regulates not only presynaptic NMDARs in the dorsal horn of the spinal cord but also postsynaptic AMPARs through its C-terminal region, promoting the clustering of NMDARs and AMPARs at synapses and thereby enhancing their expression and transport capacity (Deng et al., 2019b; Li et al., 2021). Both animal models and analyses of a limited number of human tissue samples have confirmed that the overexpression of α2δ-1 is closely associated with the development of neuropathic pain (NP) (Fujimura, 2024). Upregulation of α2δ-1 expression leads to abnormal clustering of presynaptic NMDARs and postsynaptic AMPARs, thereby increasing Ca^2+^ permeability, amplifying excitatory signals, and ultimately inducing central sensitization (Chen et al., 2018; Rodríguez-Muñoz et al., 2021). Gabapentin-like drugs can effectively block the excessive clustering of presynaptic NMDARs and postsynaptic AMPARs in the dorsal horn of the spinal cord, reduce Ca^2+^ permeability, and thereby effectively alleviate central sensitization (Varadi, 2024). In addition, the Glutamate ionotropic receptor delta type subunit 1 (GluD1) on the postsynaptic membrane of the dorsal horn of the spinal cord, together with cerebellin 1 (Cbln1) stored on the presynaptic membrane, forms a transsynaptic signaling complex that participates in pain modulation. Intravenous injection of recombinant Cbln1 restores GluD1-related synaptic signaling, thereby effectively alleviating central sensitization (Sabnis et al., 2024).

### 4.3 Central sensitization: bidirectional regulation of glutamate in brain neural circuits

The onset and maintenance of NP are closely associated with the excessive excitation of glutamatergic neurons or an abnormal increase in glutamate release within specific brain regions of the central nervous system. However, glutamatergic neurons in different brain regions may exert opposing effects—either promoting or alleviating pain—a phenomenon primarily mediated by the complexity of the neuronal networks in these regions. The brain is the most mysterious and complex organ in the human body, containing approximately 8.6 × 10^10^ neurons. These neurons not only regulate the functions of various brain regions but also interconnect via synapses to form neural circuits, thereby constituting a vast and intricate neural network (Lerner et al., 2016). The functions regulated by the brain’s neural circuits encompass a wide range of areas, including sensation, emotion, cognition, memory and motor control.

The anterior cingulate cortex (ACC) is a key brain region involved in pain perception, emotional regulation and chronic pain. In this region, astrocytes induce the release of glutamate, thereby increasing extracellular glutamate concentrations, which in turn activates presynaptic KARs, enhances synaptic transmission and consequently exacerbates central sensitisation in NP. Targeted inhibition of the expression of mGluR5 in astrocytes effectively protects ACC pyramidal neurons from glutamate transmission caused by excessive astrocyte activation, producing a significant analgesic effect (Shen et al., 2025). Caveolin-1 (Cav-1), as a structural protein of lipid rafts, is extensively involved in signal transduction and membrane protein transport (Jang et al., 2025). Studies have shown that upregulating Cav-1 expression in the ACC exacerbates pain sensitisation by enhancing glutamate release. Furthermore, cortico-cortical connections exist between the bilateral ACCs, and the glutamatergic connections between the ACCs facilitate pain perception. Activation of this pathway promotes pain perception and exacerbates central sensitisation, whilst inhibition of this pathway produces an analgesic effect (Yang J. X. et al., 2023). This suggests that glutamatergic connections between the ACCs may represent a potential therapeutic target for central sensitisation in NP. This finding provides a new cortico-cortical positive feedback mechanism for the bilateral transmission and chronicisation of NP. Inhibiting the excitability of glutamatergic neurons in the hindlimb region of the primary somatosensory cortex (S1HL) significantly reduces the firing rate of glutamatergic neurons in the S1HL region, producing an analgesic effect on NP (Qi et al., 2025). Recent studies have shown that the N6,2’-O-dimethyladenosine (m6Am) mRNA modification catalysed by phosphorylated CTD-interacting factor 1 (PCIF1) is a key trigger for the development of NP. SERPINE1 mRNA binding protein 1 (SERBP1) acts as a cofactor for PCIF1; the SERBP1-PCIF1 complex formed by the two triggers m6Am modification, thereby enhancing the excitability of glutamatergic neurons in the S1HL and exacerbating central sensitisation. Blocking the upregulation of SERBP1-PCIF1 in the S1HL can suppress the excitability of glutamatergic neurons, thereby producing an analgesic effect (Huang et al., 2025).

The ventrolateral periaqueductal grey (vlPAG) and the lateral parabrachial nucleus (LPBN) of the midbrain are key centres regulating NP; glutamatergic neurons in the vlPAG and LPBN play distinctly different roles in the transmission of pain signals. Specifically, activation of glutamatergic neurons within the vlPAG effectively alleviates pain centre sensitisation, whilst inhibition of glutamatergic neuron activation within the LPBN exerts an analgesic effect on NP (Sun et al., 2020). It is worth noting that the activity of glutamatergic neurons within the LPBN is also regulated by Gamma-aminobutyric acid (GABA) neurons (Zhu et al., 2024). GABAergic neurons within the LPBN alleviate central sensitisation by directly inhibiting local glutamatergic neurons. Recent studies have shown that the activity of glutamatergic neurons within the LPBN is under the control of glutamatergic projections from the spinal trigeminal nucleus caudalis (Sp5C), leading to sustained pain sensitisation (Zhang et al., 2021). The inhibition of glutamatergic neuron activity within the vIPAG is another important factor in inducing central sensitisation in NP. Inhibition of astrocytes in the ventrolateral orbitofrontal cortex (vlOFC) using optogenetic techniques can restore the vlOFC–vIPAG glutamatergic descending pain regulatory pathway, thereby further suppressing abnormal excessive discharge in the ventral posteromedial nucleus (VPM) and producing a significant analgesic effect on NP (Islam et al., 2025b).

The central sensitization is also accompanied by an increase in glutamate release in the thalamus. Specifically, activation of glutamatergic neurons in the paraventricular thalamic nucleus (PVT) leads to elevated levels of transient receptor potential cation channel subfamily member 6 (TRPC6), which in turn induces Ca^2+^ influx to stimulate and activate PKC, thereby triggering pain sensitization (Jiang et al., 2026). Electroacupuncture treatment can significantly inhibit TRPC6 activation, reduce calcium influx, lower PKC phosphorylation levels, and produce an analgesic effect by reducing the release of the excitatory neurotransmitter glutamate within the PVT. As a key relay station for pain signal transmission, the thalamus plays a central role in the central mechanisms of pain regulation. Abnormalities in neurotransmitters and alterations in functional connectivity within the central nervous system are important mechanisms underlying NP. Increased glutamate release in the thalamus promotes enhanced thalamic–insular functional connectivity by enhancing the activity of excitatory neurons, thereby further exacerbating NP (Wang et al., 2020). Glutamate in the medial area of secondary visual cortex (V2M), as a key component of the endogenous pain regulation network, becomes hyperactive following nerve injury, thereby promoting NP and associated negative emotions. In a tracer study utilising neuronal tracing techniques to examine V2M in mice with partial sciatic nerve ligation (PSL), it was demonstrated that V2M glutamate makes dense synaptic projections to the lateral posterior thalamic nucleus (LP) and the lateral dorsal thalamic nucleus (LD); however, only V2M glutamate projections to the LP promote the transmission of pain signals (Tan et al., 2025). This suggests that targeted inhibition of the V2M glutamate–LP pathway may be a potential therapeutic strategy for alleviating central sensitisation in NP. Furthermore, in spared nerve injury (SNI) model mice, synaptic transmission by glutamatergic neurons in the ventral tegmental area (VTA) is attenuated. Optogenetic activation of VGLUT3-expressing neurons in the dorsal raphe nucleus (DRN) projecting to the VTA significantly inhibits pain sensitisation (Wang et al., 2023). This suggests that the glutamate-mediated DRN–VTA circuit plays a regulatory role in central sensitisation associated with NP. Neurons in the nucleus tractus solitarius (NTS) integrate sensory signals from the viscera and the body, and then transmit this information to other brain regions (Roman et al., 2017; Ma et al., 2019). When NP occurs, glutamate activity in the rostral ventrolateral medulla (RVLM) of the medulla oblongata is enhanced, contributing to the maintenance of sympathetic excitation (Suzuki et al., 1997). Studies have shown that inhibiting the RVLM–NTS projection mediated by glutamatergic neurons effectively reduces sympathetic excitability, producing a significant analgesic effect on NP (Chen et al., 2025).

## 5 Discussion and conclusions

For centuries, the origins of pain and its dual role as both a key physiological function and a debilitating condition have captivated countless scientists and philosophers. From Descartes’ assertion that ‘pain is transmitted from a specific point in the body to the brain via specific pathways’ to the formulation of the Melzack–Wall pain gate control theory, human understanding of the nature of pain has undergone a profound shift from mechanical reductionism to a multidimensional, integrative perspective. The IASP defines pain as ‘an unpleasant sensory and emotional experience associated with, or resembling, actual or potential tissue damage’. This landmark definition not only acknowledges the subjectivity and multidimensional nature of pain, but also fundamentally challenges the traditional view that ‘tissue damage inevitably leads to pain’. Subsequently, the 2019 ICD-11 classified chronic pain as a distinct disease, marking the formal transition of pain medicine from being regarded as a ‘symptom’ to being recognised as a ‘disease’. However, new questions arising from this transition still require resolution. Namely, when pain is recognised as a disease, what exactly is its aetiology? And how is pain caused by underlying tissue damage to be determined? These questions not only concern the theoretical framework of basic research but also profoundly influence clinical diagnosis, the selection of treatment strategies and patients’ social recognition.

The glutamate system occupies an irreplaceable central position in research into the pathophysiological mechanisms of NP. This review systematically summarises the multidimensional regulatory roles of glutamate and its receptors in the pain pathways of NP in recent years, revealing a complex regulatory network spanning from the molecular to the circuit level, and from the periphery to the central nervous system. At the peripheral level, α2δ-1 in the DRG mediates NMDAR activity, leading to abnormal glutamate excitation, which in turn amplifies glutamatergic signals from Aδ fibres and ultimately induces pain sensitization. At the central level, the downregulation of EAAT2 expression in the dorsal horn of the spinal cord, the abnormal upregulation of VGLUT1/2 expression, and the excessive clustering of α2δ-1 with NMDARs and AMPARs at synapses all act synergistically to drive the onset and maintenance of central sensitisation. Furthermore, at the level of central brain regions, the transmission of pain signals by glutamatergic neurons is more complex. Specifically, activating glutamatergic neurons within the vlPAG effectively alleviates central sensitization, while inhibiting the activation of glutamatergic neurons in the LPBN exerts an analgesic effect. This specific functional differentiation of glutamatergic neurons across different brain regions suggests that simply enhancing or inhibiting the glutamatergic system in a single brain region may prove ineffective in alleviating pain. In future, it will be necessary to combine techniques such as neuronal tracing, optogenetics and chemogenetics to conduct in-depth research into the specific mechanisms by which glutamatergic neurons mediate pain signal transmission across different brain regions, thereby optimising brain-region-targeted intervention strategies. It is worth noting that recent research has revealed a key mechanism: central sensitisation in NP is not only the result of enhanced glutamatergic excitability, but also a ‘disinhibition’ effect caused by impaired function of inhibitory neurotransmitters. In other words, abnormal excitation of the glutamatergic system and weakened GABAergic inhibition constitute an inseparable pathological process. This suggests that simply blocking glutamate and its receptors may not fully alleviate pain; instead, enhancing inhibitory input or restoring the excitation-inhibition balance should be pursued simultaneously, which may yield superior analgesic effects.

Currently, research into NP focuses primarily on the following three areas: (1) Identifying potential translational mechanisms: how acute pain transitions to chronic pain by influencing pain perception, spinal processing and pain modulation in the brain; (2) Quantifying susceptibility or risk: identifying new diagnostic or predictive biomarkers and refining existing ones, thereby objectively quantifying pain, identifying the risk of chronic pain following injury, and predicting outcomes; (3) Identifying candidate treatment strategies: based on large-scale databases and multicentre clinical trials, constructing an Intervention Variability Evaluation (IVE) predictive model encompassing the three pillars of intervention, patient variability and outcome evaluation, and establishing personalised treatment algorithms (Cohen et al., 2021). However, animal experiments remain an indispensable fundamental tool for elucidating pain neural circuits and molecular targets (Cao et al., 2024). It is precisely through animal models that researchers have been able to elucidate core pain transmission mechanisms, such as peripheral and central sensitisation, thereby advancing the development of standard treatment protocols for NP centred on pharmacotherapy, behavioural therapy and interventional therapies (Wang and Doan, 2024). However, existing animal models struggle to replicate the cognitive and emotional dimensions associated with clinical pain, and there is a significant gap between pain-related behaviour in rodents and subjective pain perception in humans. Furthermore, there is an imbalance in the application of omics technologies in animal experiments (excessive reliance on the microbiome and metabolome, insufficient integration of single-cell transcriptomics, spatio-omics and proteomics, and limited depth of data analysis). These factors not only hinder the development of novel analgesics but also significantly reduce the efficiency of translating findings from animal experiments into clinical research (Mao, 2012).

Research into the glutamate system in the field of NP still faces several pressing issues. Firstly, the spatiotemporal dynamics of glutamate signalling remain unclear. The onset, maintenance and chronicity of NP constitute a dynamically evolving process. However, almost all current studies rely on static molecular expression assays or *in vitro* electrophysiological recordings, with a lack of research into glutamate release, receptor aggregation and clearance at different stages of the disease. The development of *in vivo*, real-time, high-resolution techniques for monitoring glutamate dynamics will be key to resolving this issue. Second, there is a significant disconnect between peripheral and central sensitization. The transmission of pain signals between the spinal cord and brain regions remains to be clarified; future research should incorporate techniques such as neuronal tracing to systematically investigate the pain-modulating networks between brain regions and between the brain and spinal cord. Finally, the mechanisms of cross-talk between the glutamate system and the neuroimmune system remain a relatively under-explored area. The activation of microglia and astrocytes not only affects glutamate clearance but also regulates synaptic plasticity through the release of inflammatory factors. It is recommended that future research utilise tools such as Microbe of Traditional Chinese Medicine to explore multi-target synergistic mechanisms for the treatment of NP from the perspective of the neuroimmune axis.

In summary, the glutamate system plays an irreplaceable and central role in the development and maintenance of NP. From abnormal discharges in the DRG to the excitation–inhibition imbalance in the dorsal horn of the spinal cord, and on to the bidirectional regulation of circuits in multiple brain regions, every stage of this process is inextricably linked to the glutamate system. Glutamate is involved in the encoding, transmission, amplification and perception of pain signals in a multidimensional manner. It is precisely for this reason that analgesic strategies targeting the glutamate system face significant challenges.

## Glossary

AMPAR: α-amino-3-hydroxy-5-methyl-4-isoxazolepropionic acid receptor
ACC: anterior cingulate cortex
Cbln1: cerebellin 1
Cav-1: Caveolin-1
DRG: dorsal root ganglion
DRN: dorsal raphe nucleus
EAAT: Excitatory Amino Acid Transporter
EAAT2: excitatory amino acid transporter 2
GluN1: Glutamate ionotropic receptor NMDA type subunit 1
GluD1: Glutamate ionotropic receptor delta type subunit 1
GABA: Gamma-aminobutyric acid
IASP: International Association for the Study of Pain
KAR: Kainate receptor
LPBN: lateral parabrachial nucleus
LP: Lateral Posterior Thalamic Nucleus
LD: lateral dorsal thalamic nucleus
mGluR: Metabotropic glutamate receptor
MOR: Mu-opioid receptor
mGluR5: Metabotropic glutamate receptor 5
m6Am: N6,2’-O-dimethyladenosine
NP: neuropathic pain
NMDAR: N-methyl-D-aspartate receptor
NTS: nucleus tractus solitarius
PHN: postherpetic neuralgia
PSL: Partial Sciatic Nerve Ligation
PCIF1: phosphorylated CTD-interacting factor 1
PVT: Paraventricular nucleus of the thalamus
PKC: protein kinase C
RVLM: rostral ventrolateral medulla
S1HL: primary somatosensory cortex hindlimb region
SERBP1: SERPINE1 mRNA binding protein 1
Sp5C: spinal trigeminal nucleus caudalis
SNI: spared nerve injury
TRPC6: transient receptor potential cation channel subfamily C member 6 Gene
VGLUT: Vesicular Glutamate Transporter
VGCC: voltage-gated calcium channel
VGLUT2: vesicular glutamate transporter 2
VGLUT1: vesicular glutamate transporter 1
vlPAG: ventrolateral periaqueductal grey
vLOFC: ventrolateral orbitofrontal cortex
VPM: Ventral posteromedial nucleus
V2M: medial area of secondary visual cortex
VTA: ventral tegmental area
WDR: wide dynamic range
α2δ-1: calcium voltage-gated channel auxiliary subunit alpha2delta 1
